# Intrathecal silymarin administration improves recovery after compression spinal cord injury: evidence for neuroprotection, antioxidant, and anti-inflammatory action

**DOI:** 10.3389/fphar.2025.1592682

**Published:** 2025-08-29

**Authors:** Omar Bahmani, Amir Kiani, Sajad Fakhri, Fatemeh Abbaszadeh, Khodabakhsh Rashidi, Javier Echeverría

**Affiliations:** ^1^ Student Research Committee, Kermanshah University of Medical Sciences, Kermanshah, Iran; ^2^ Regenerative Medicine Research Center, Kermanshah University of Medical Sciences, Kermanshah, Iran; ^3^ Pharmaceutical Sciences Research Center, Health Institute, Kermanshah University of Medical Sciences, Kermanshah, Iran; ^4^ Neurobiology Research Center, Institute of Neuroscience and Cognition, Shahid Beheshti University of Medical Sciences, Tehran, Iran; ^5^ Research Center of Oils and Fats, Kermanshah University of Medical Sciences, Kermanshah, Iran; ^6^ Departamento de Ciencias del Ambiente, Facultad de Química y Biología, Universidad de Santiago de Chile, Santiago, Chile

**Keywords:** spinal cord injury, silymarin, neuropathic pain, motor function, oxidative stress, neuroprotection, inflammation

## Abstract

**Background:**

Spinal cord injury (SCI) is a debilitating disorder that affects people’s quality of life. Unfortunately, there is no definitive drug for treating SCI. Additionally, the adverse effects of existing non-approved drugs make it necessary to research and investigate the effects of new multi-target agents to combat SCI complications.

**Purpose:**

This study specifically investigates the effect of a multi-target phytochemical silymarin (SIL), known for its potent neuroprotective, anti-inflammatory, and antioxidant properties, on sensorimotor function after SCI.

**Materials and Methods:**

In total, 30 male Wistar rats were divided into five distinct groups: Sham, SCI, and three additional groups that received SIL at dosage levels of 0.1, 0.2, and 0.4 μmol. Following the injury, behavioral tests such as acetone drop, hot plate, von Frey, BBB, and inclined plane were conducted along with weight measurements for 4 weeks. Serum samples were analyzed to assess alterations in catalase and glutathione levels, nitrite concentration, and the activity of matrix metalloproteinases (MMP) 2 and 9. Besides, histopathological studies were done to evaluate the number of neurons in the spinal cord tissue.

**Results and discussion:**

Various doses of SIL, particularly the 0.2 μmol dosage, significantly influenced the alleviation of pain, enhancement of motor function, and weight gain in animals following SCI. In addition, SIL increased the levels of catalase and glutathione, while decreasing serum nitrite levels. It also increased anti-inflammatory MMP2 levels and the sensory/motor neurons’ survival, while decreasing inflammatory MMP9.

**Conclusion:**

Generally, intrathecal injection of SIL after SCI provides neuroprotective, anti-inflammatory, and antioxidant effects leading to pain reduction and improved motor function in rats.

## 1 Introduction

Spinal cord injury (SCI) is a debilitating condition that disrupts communication between the brain and body, affecting sensation, movement, and autonomic functions. It can result from both traumatic incidents, such as car accidents and falls, and non-traumatic causes like infections, tumors, or blood clots (Alizadeh et al., 2019). The primary mechanism of SCI occurs at the time of initial trauma and involves mechanical disruption of the spinal cord tissue. Secondary mechanisms of SCI refer to ongoing cellular and molecular processes that occur after the primary injury. These secondary mechanisms can lead to further damage and worsening of the primary injury, potentially leading to long-term neurological deficits. Inflammation, edema, excitotoxicity, ischemia, axonal injury, apoptosis, and oxidative stress are some of the secondary mechanisms involved in SCI (Freire et al., 2023). One of the primary contributors to neuronal damage post-SCI is oxidative stress, characterized by the excessive production of reactive oxygen species (ROS) such as superoxide and hydrogen peroxide. These ROS can inflict cellular damage by oxidizing proteins, lipids, and DNA, ultimately leading to cell death through apoptosis or necrosis (Fakhri et al., 2022b). Increased expression of inducible nitric oxide synthase (iNOS) has been observed in injured spinal cord tissues, correlating with elevated levels of nitric oxide (NO) and subsequent formation of peroxynitrite, a potent oxidant that further damages cellular structures (Kwak et al., 2005). In addition to oxidative stress, the inflammatory response plays a critical role in SCI pathology. Following injury, there is an activation of resident immune cells such as microglia and astrocytes, alongside infiltration of peripheral immune cells like neutrophils and macrophages. The activation of the immune system triggers the release of pro-inflammatory cytokines (e.g., TNF-α, IL-1β) and chemokines that can exacerbate tissue damage through sustained inflammation and recruitment of additional immune cells to the injury site (Gensel and Zhang, 2015; Liu et al., 2021). Matrix metalloproteinases (MMPs), particularly MMP2 and MMP9, are critical mediators of inflammation following SCI (Zhang et al., 2011). As previously described, while MMP9 plays an inflammatory role, MMP2 possesses anti-inflammatory potential in the context of SCI (Bagheri Bavandpouri et al., 2024).

Considering the complexity of the SCI pathophysiology, unfortunately, there is no effective cure for SCI. On the other hand, the adverse effects of non-approved drugs used for SCI make it crucial to explore new multi-target medications addressing the complex nature of SCI and providing potential therapeutic benefits. Natural phenolic compounds are hopeful sources of multi-targeting agents with a successful history of neuroprotection. Silymarin (SIL), derived from the seeds of milk thistle (*Silybum marianum* (L.) Gaertn. [Asteraceae]), is a rich source of flavonolignans like silybin, silychristin, and silydianin. These compounds exhibit notable antioxidant and anti-inflammatory characteristics (AbouZid et al., 2016). SIL has been used for centuries as an herbal remedy for various liver-related conditions (Singla et al., 2024). It is believed to protect liver cells from damage, promote the regeneration of liver tissue, and improve liver function (Gillessen and Schmidt, 2020). SIL has also been noted for its potential protective effects, such as cancer prevention (Koltai and Fliegel, 2022), type 2 diabetes mellitus management (Hadi et al., 2018), and improvement of nervous system disorders (Guo et al., 2019). Some possible neuroprotective mechanisms of SIL have been reported through scavenging free radicals and enhancing the activity of antioxidant enzymes (Surai, 2015). It has been reported that SIL inhibits the expression and production of pro-inflammatory cytokines such as tumor necrosis factor (TNF-α) and interleukin-1beta (IL-1β), which are involved in the pathogenesis of neurodegeneration (Ullah and Khan, 2018). In addition, reports indicated that SIL activated the protein kinase B (Akt)/mammalian target of rapamycin (mTOR) signaling pathway, as well as suppressed the inflammatory nuclear factor-kappa B (NF-κB) while simultaneously enhancing the expression of the anti-apoptotic Bcl-2 (Wang et al., 2012). In a previous report, SIL showed anti-inflammatory effects in a different SCI model, contusion or weight drop method, with a limited behavioral test. They were more focused on *in vitro* results to confirm the protective role of SIL on spinal cord cortical cells against oxidative stress and lipopolysaccharide stimulation (Tsai et al., 2010).

According to the aforementioned antioxidant and anti-inflammatory roles of SIL in neurodegeneration and other diseases, as well as its multi-targeting potentials, the current study is the first to examine the impact of intrathecal (i.t.) administration of SIL in the regulation of sensory and motor dysfunction after SCI, employing multiple behavioral tests with evidence on its neuroprotection, antioxidant, and anti-inflammatory effects.

## 2 Materials and methods

### 2.1 Chemicals and reagents

Silymarin and Coomassie blue were purchased from Sigma-Aldrich (St. Louis, MO, United States), and cefazolin from Exir Company (Boroujerd, Iran). Tris Base and SDS were prepared from Merck Company (Darmstadt, Germany). Triton™ X-100 was purchased from Acros Organics (Geel, Belgium), and CaCl_2_ was purchased from Merck Company (Darmstadt, Germany). All the other chemicals and reagents were of analytical reagent grade, purchased from commercial sources.

### 2.2 Experimental animals

A total of 30 adult male Wistar rats weighing between 230 and 250 g were housed in the animal house of the Kermanshah University of Medical Sciences. Rats were randomly assigned to five groups using a lottery. Rats were housed in cages with randomized cage placement across shelves to avoid any rack effects (e.g., light, temperature gradients). Animals were housed in polypropylene cages with wood shavings bedding under a 12 h light/dark cycle (lights on 7:00 a.m. to 7:00 p.m.), maintained at 22 °C ± 2 °C and 50% ± 10% humidity, and provided *ad libitum* access to standard chow and water.

### 2.3 Spinal cord injury and animal groups

The rats were administered ketamine and xylazine (80/10 mg/kg) to induce deep anesthesia. A laminectomy procedure was then performed at the thoracic (T8-T9) level using a micro rongeur. After that, an aneurysm clamp with a force of 90 g was used for 1 min to apply severe SCI in the SCI and SIL groups (*n* = 6) (Huang et al., 2007; Zhang et al., 2019; Jiang et al., 2023; Amirian et al., 2025). After surgery, the tissue and skin were sutured. In addition, cefazolin (40 mg/kg, intraperitoneal) and normal saline (2 mL, subcutaneous) injections and bladder massage were carried out manually twice daily until normal bladder function was regained (Fakhri et al., 2018; 2019). The rats were assigned to five groups at random: a Sham group, an SCI group, and three groups that received varying doses of SIL (0.1, 0.2, and 0.4 μmol). About 30 min after surgery/compression injury, the sham and SCI group received vehicle, and three treatment groups received 10 μL of SIL at doses of 0.1, 0.2, and 0.4 μmol dissolved in the same vehicle.

Regarding preparing vehicle or SIL for i.t. injection, first, SIL was dissolved in 70% ethanol to prepare the stock solution (Tsai et al., 2010; Javed et al., 2011; Csupor et al., 2016). Then, this solution was diluted with distilled water at a ratio of 1–9 (1/9: solution/distilled water: v/v) to achieve the desired concentration for injection. Finally, a 10 μL volume of this diluted solution was selected and used for i.t. injection. Exclusion criteria were dying animals during surgery/protocol or a rare failure in the i.t. injection.

### 2.4 Intrathecal drug injection

Mester’s modified method was used for i.t. injection of 10 μL of vehicle or SIL. Briefly, a 25-gauge needle connected to Hamilton was inserted at a 45° angle into the space between the two lumbar parts of L5 and L4. After observing a sudden tail reflex, the injection was done slowly for 10 s (Mestre et al., 1994).

### 2.5 Weight change

The rats’ weights were measured weekly starting from the beginning of the study. To calculate the changes in weight for each group, the following formula was used:

Weight changes = (weight on days post-surgery-weight on day 0).

### 2.6 Behavioral tests

Behavioral tests (i.e., BBB scale, inclined plane test, thermal and mechanical allodynia assessments) were selected to evaluate both motor and sensory functional recovery following SCI comprehensively. The BBB scale and inclined plane test assess locomotor ability and balance, which are directly impaired after spinal cord trauma and are central outcomes for neuroprotection studies. Thermal hyperalgesia and cold/mechanical allodynia tests were utilized to quantify alterations in sensory function and neuropathic pain symptoms, which are critical secondary complications of SCI. Behavioral assessments were performed on all animals both before surgery (day 0) and weekly post-surgery for 28 days.

#### 2.6.1 Basso, beattie, and bresnahan (BBB) test

The BBB scale is a locomotor rating scale. The animals’ movements were observed in a 100 × 100 × 10 cm box for 5 min. The BBB scale scores ranged from 0 (no observable hindlimb movement) to 21 (normal locomotion). The final score was determined by averaging the scores for both hindlimbs (Fakhri et al., 2021).

#### 2.6.2 Inclined plane test

The ability of rats’ legs to bear body weight and stand on a ramp was assessed using a wooden plane measuring 60 × 40 cm. The wood plane is inclined at angles ranging from 0° (horizontal) to 60°. The maximum angle at which rats were able to sustain their position for 5 s was recorded (Samini et al., 2013).

#### 2.6.3 Hot plate test

Heat hyperalgesia was assessed by applying thermal stimulation to the hind paw of the animal. Rats were placed in a transparent chamber on a hot plate with temperatures between 48 °C and 52 °C. Pain-related behaviors, such as paw biting or licking, were noted as paw withdrawal latency (PWL). A cut-off time of 60 s was implemented to stop the thermal stimulation (Fakhri et al., 2022c).

#### 2.6.4 Von frey test

To assess mechanical allodynia, the von Frey test was employed using incremental forces measured in 10, 15, 26, 60, and 100 g (Stoelting, IL, United States). Each filament was applied to the sole five times, with a 5-s interval between each application. The presence of behaviors such as hindpaw withdrawal, flinching, licking, or biting was considered a positive response indicating allodynia. To determine the allodynia threshold, a positive response in at least three out of the five repeated stimuli was used as the criterion (Fakhri et al., 2019).

#### 2.6.5 Acetone drop test

The effects of acetone-induced cold stimulation on the paw of rats were assessed by applying 0.1 mL of acetone from a distance of 2 cm. The rats’ response was evaluated based on their hind claw reaction, and the results were classified and given scores according to the following categories: no paw withdrawal (0), startle response without paw withdrawal (1), slight paw withdrawal (2), prolonged paw withdrawal (3), licking and flinching (4) (Kauppila, 2000; Fakhri et al., 2018).

### 2.7 Histological analysis

Neuron counting in dorsal and ventral horns provides direct evidence of neuronal preservation or loss post-injury and treatment, reflecting neuroprotective effects of SIL at the cellular level.

On day 28, animals were perfused with normal saline and then 4% paraformaldehyde. A piece of spinal cord was then dissected at the center of the lesion site (8th vertebra), fixed in paraffin, and 7-μm sections were prepared according to established protocols. Slides from each section were stained using hematoxylin and eosin (H&E). Images were captured at a magnification of 40x, and the neuron count in both the dorsal and ventral horns was assessed utilizing ImageJ software (NIH). The neuron count was expressed as a percentage relative to the control group (Fakhri et al., 2019).

### 2.8 Zymography

Given the known roles of MMPs in SCI-related inflammation and repair, assessing MMP2 and MMP9 levels allowed us to investigate SIL’s modulatory effects on inflammatory and anti-inflammatory pathways, correlating with functional recovery.

On the 28th day, MMP2 and MMP9 gelatin zymography was conducted using 7.5% SDS-PAGE gels copolymerized with 0.1% gelatin. Each sample, obtained from rat aorta on the 28th day, was loaded with a total serum protein of 100 μg, determined using the Bradford assay. Electrophoresis was carried out using the Biorad Mini Protean III mini-gel slab device at a constant voltage of 150 V. After washing the gels in a buffer containing Triton X-100 and Tris-HCl (pH 7.5), incubation was performed in a buffer consisting of 10 mM CaCl_2_, 0.15 M NaCl and 0.02% NaN_3_ in 50 mM Tris-HCl (pH 7.5) for 18 h at 37 °C. Following the incubation, the gels were stained with Coomassie Blue and then destained using a solution composed of 5% acetic acid and 7% methanol. Enzymatic activity appeared as clear bands on the blue background. Specific band intensities were relatively calculated using ImageJ software (Fakhri et al., 2021).

### 2.9 Biochemical assays: nitrite, catalase, and glutathione

These markers were chosen to evaluate oxidative stress and nitrosative damage, key secondary injury mechanisms in SCI. Catalase and glutathione represent antioxidant defense, while nitrite levels serve as an indicator of NO-mediated oxidative injury. These assays align with SIL’s proposed antioxidant role and help elucidate its mechanism of action.

#### 2.9.1 Catalase activity assay

The Aebi method was used to assess the catalase activity (Aebi, 1984). Briefly, 20 μL of rat serum was added to each well plate, followed by 100 μL of a 65 mM hydrogen peroxide solution, and incubated at 25 °C for 4 min. The reaction was stopped by adding 100 μL of ammonium molybdate, and the mixture was then measured at 405 nm using an ELISA reader.

#### 2.9.2 Glutathione activity assay

The glutathione level was measured using Ellman’s method in this study (Eyer and Podhradský, 1986). To perform the measurement, 20 μL of rat serum, obtained from rat aorta on the 28th day, was added to the wells of a 96-well plate. Subsequently, 50 μL of phosphate buffer with a pH of 7 was added to each well. Then, 40 μL of dithiobisnitrobenzoic acid (DTNB) was added to the mixture. The plate was incubated for 10 min at 37 °C. Finally, the absorbance of the solution was read at a wavelength of 412 nm using an ELISA reader (Eyer and Podhradský, 1986). To calculate the absorption difference between the treatment groups and the sham group, the following formula was used:

Percentage difference = ((C_sham_–C_sample_)/C_sham_) x 100.

The C_sham_ is the amount of catalase or glutathione in the samples of the Sham group, and the C_sample_ is the amount of catalase or glutathione in the samples of other groups, including SCI or SIL-treated groups (Fakhri et al., 2022c).

#### 2.9.3 Nitrite assay

According to the Griess reaction assay (Sun et al., 2003), to prepare the serum samples for analysis, protein removal was carried out by adding zinc sulfate. After that, the samples, obtained from the rat aorta on the 28th day, were centrifuged. The supernatant was mixed with vanadium chloride solution in a one-to-one ratio. Following, a Griess solution containing sulfanilamide (2%) and 0.1% diamide dihydrochloride was added to the samples. The samples were then incubated for 30 min at a temperature of 37 °C. After the incubation period, the resulting color was measured at a wavelength of 540 nm using a spectrophotometer. The absorbance of the samples was calculated by comparing it to the absorbance of a standard reference, and the concentration of nitrite in the samples was determined (Sun et al., 2003).

**FIGURE 1:**
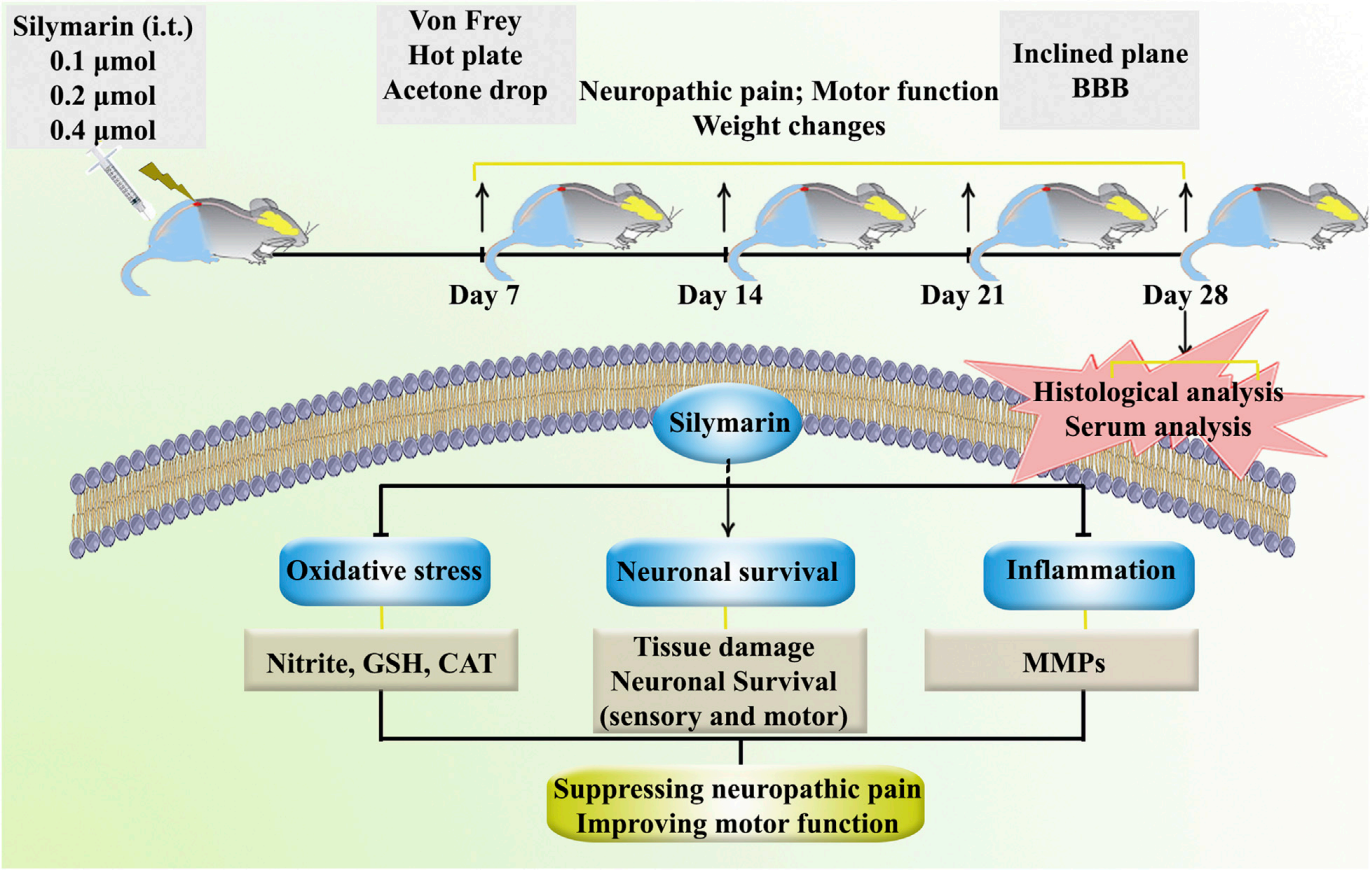
A summary of the research protocols and the regulatory role of SIL against neuropathic pain and motor dysfunction through tissue-protective, anti-inflammatory, and antioxidant effects.

Figure 1 presents an overview of the research protocols (Figure 1).

### 2.10 Statistical analysis

Data are presented as mean ± standard error of the mean (SEM). Statistical analyses were performed using GraphPad Prism v8. For comparison of multiple groups over time (e.g., behavioral tests, weight changes), two-way ANOVA was applied, followed by Bonferroni’s *post hoc* test to evaluate group and time effects. For single-time-point biochemical and histological measurements, one-way ANOVA was used with Tukey’s multiple comparisons *post hoc* test. The significance threshold was set at *p* < 0.05.

## 3 Results

### 3.1 Weight changes

**FIGURE 2:**
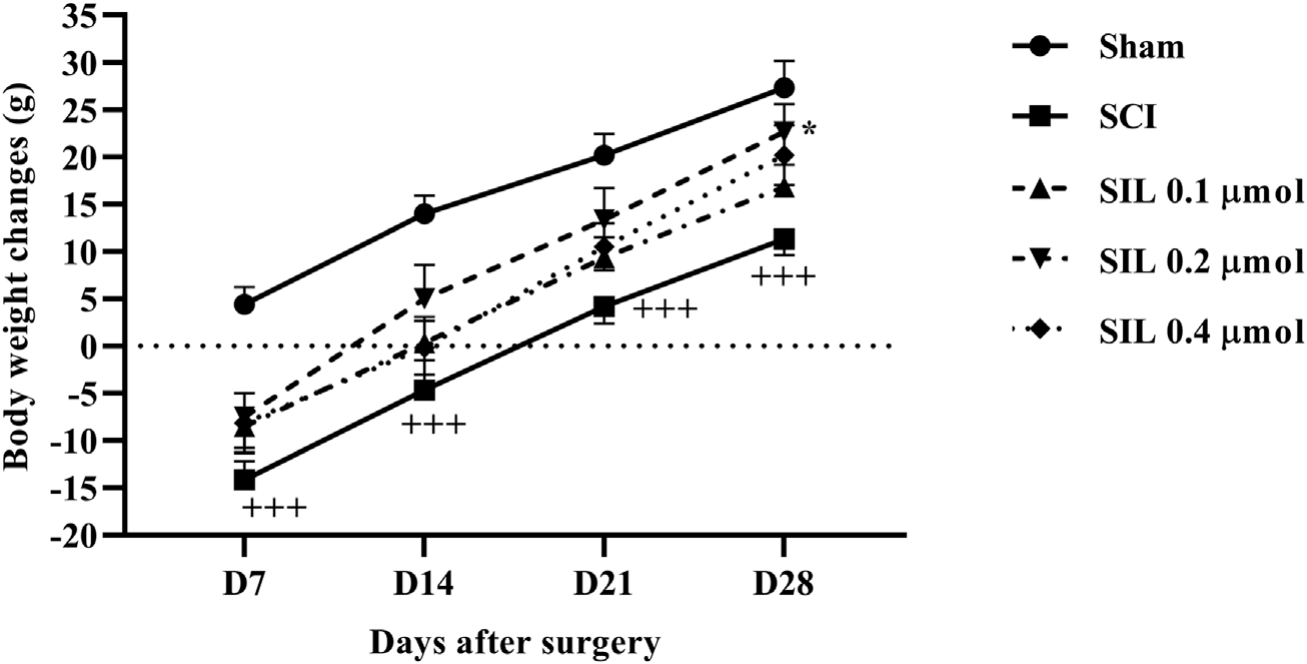
Effects of SIL on body weight changes following compression SCI. Data are expressed as mean ± SEM (*n* = 6). ^+++^ *p* < 0.001 vs. sham group and ^*^ *p* < 0.05 vs. SCI group. A two-way ANOVA was used. SCI: spinal cord injury; SIL: silymarin (0.1, 0.2, and 0.4 μmol, 10 μL, 30 min post-SCI).

During the 4-week follow-up period, the sham group exhibited a normal pattern of weight gain. However, the animals in the SCI group experienced weight loss because of SCI. Interestingly, treatment with various doses of SIL, particularly the dose of 0.2 μmol, improved the weight gain of the animal after injury (*p* < 0.05 in comparison to SCI). As a result, by the 28th day, the animals in this group were able to achieve a weight range similar to that of the sham group (Figure 2).

### 3.2 Behavioral results

#### 3.2.1 Locomotor activity

**FIGURE 3:**
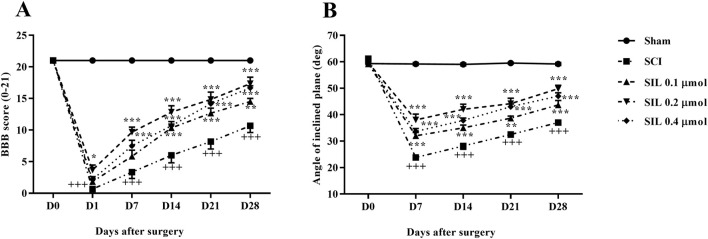
Effects of SIL on locomotor activity following compression SCI. BBB score **(A)**, inclined-plane **(B)** test. Data are expressed as mean ± SEM (*n* = 6). ^+++^ *p* < 0.001 vs. sham group and ^*^ *p* < 0.05, ^**^ *p* < 0.01, ^***^ *p* < 0.001 vs. SCI group. A two-way ANOVA was used. SCI: spinal cord injury; SIL: silymarin (0.1, 0.2, and 0.4 μmol, 10 μL, and 30 min post-SCI).

The motor behavior of the rats was assessed using the BBB scale and inclined plane test after SCI. Generally, rats in the sham group consistently scored 21, demonstrating that the laminectomy procedure did not cause any functional deficits. In contrast, rats with SCI initially had average scores of 0 and 2 on the first day post-injury, and these scores remained significantly lower than those of the sham group until day 28 post-SCI (*p* < 0.001). Administering various doses of SIL led to a marked improvement in motor function in SCI rats, beginning as early as the first day, compared to the SCI group (*p* < 0.001) (Figure 3A).

The ability to stand and maintain balance on the ramp was the same in the sham group and was approximately at an angle of 60°. However, in the group with SCI, there was a notable decline in the average angle of stay that was statistically significant compared to the sham group, ranging from 20° to 30° (*p* < 0.001). Instead, rats treated with different doses of SIL showed significant improvement from the first week (35° – 45°) (*p* < 0.001) (Figure 3B).

#### 3.2.2 Heat hyperalgesia

**FIGURE 4:**
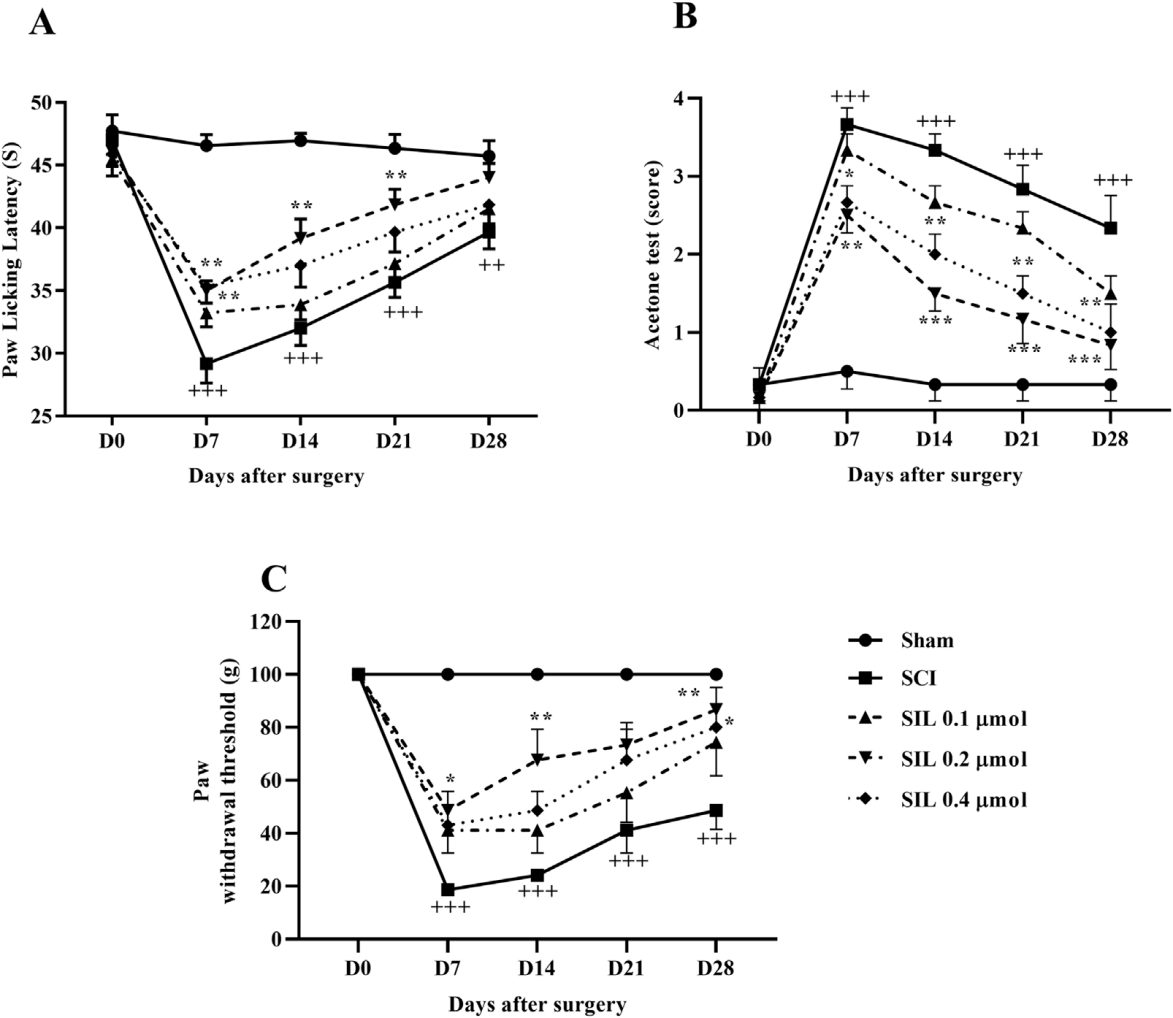
Effects of SIL on pain-related behaviors following compression SCI. Paw response threshold to thermal **(A)**, cold **(B)**, and mechanical **(C)** stimuli. Data are expressed as mean ± SEM (*n* = 6). ^++^ *p* < 0.01, ^+++^ *p* < 0.001 vs. sham group and ^*^ *p* < 0.05, ^**^ *p* < 0.01, ^***^ *p* < 0.001 vs. SCI group. A two-way ANOVA was used. SCI: spinal cord injury; SIL: silymarin (0.1, 0.2, and 0.4 μmol, 10 μL, and 30 min post-SCI).

In the sham group, paw-licking latency was almost the same on all test days. SCI was associated with a significant decrease in paw lick latency from day 7 (*p* < 0.001). Treatment with SIL, especially the dose of 0.2 μmol, significantly increased the response threshold of animals to thermal stimuli from day 7 (*p* < 0.01) (Figure 4A).

#### 3.2.3 Cold allodynia

Two-way ANOVA analysis of cold pain response data revealed that the sham group maintained normal cold hypersensitivity throughout the 28 days. In contrast, the SCI group exhibited significant hypersensitivity to cold allodynia compared to the sham group (*p* < 0.001). Additionally, various doses of SIL administered from the first week demonstrated a significant difference in response to the cold stimulation compared to the injury group (*p* < 0.001) (Figure 4B).

#### 3.2.4 Mechanical allodynia

Although the sham group exhibited a similar paw withdrawal response throughout the 4-week follow-up period, SCI resulted in a significant and persistent decrease in the paw withdrawal threshold (*p* < 0.001), indicating the presence of mechanical allodynia. Notably, treatment with SIL, especially the dose of 0.2 μmol, effectively attenuated the symptoms induced by SCI from day 7 (*p* < 0.001) (Figure 4C).

### 3.3 Histological analysis

**FIGURE 5:**
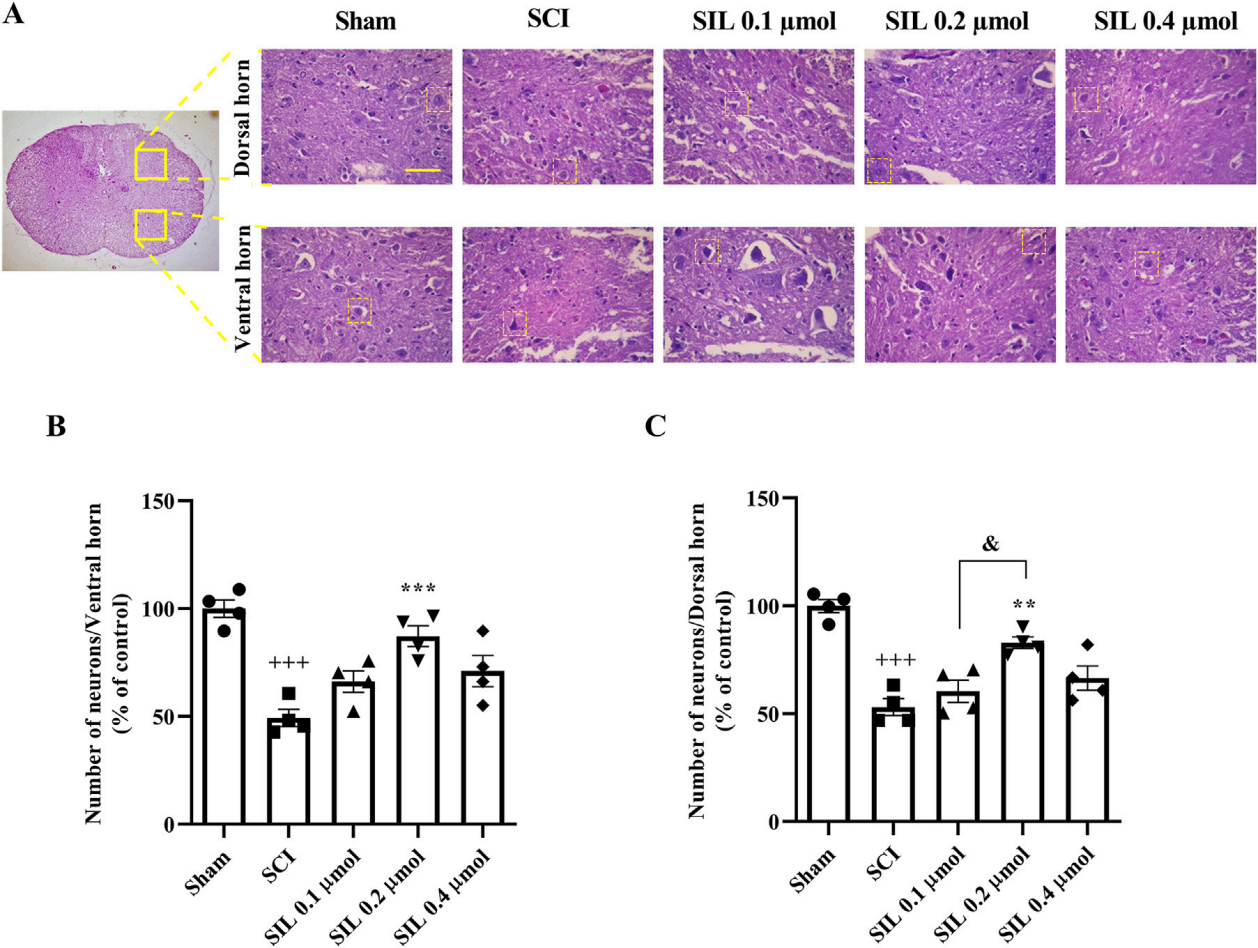
Effects of SIL on the number of neurons of the spinal cord following compression SCI **(A)**, ventral **(B)**, and dorsal **(C)** horns. Data are expressed as mean ± SEM (*n* = 4). ^+++^ *p* < 0.001 vs. sham group and ^**^ *p* < 0.01, ^***^ *p* < 0.001 vs. SCI group and ^&^ *p* < 0.05 vs. SIL 0.2 μmol group. A one-way ANOVA was used. SCI: spinal cord injury; SIL: silymarin (0.1, 0.2, and 0.4 μmol, 10 μL, and 30 min post-SCI). The yellow line in the figure shows 100 μm.

Figure 5A displays sections stained with H&E. Neurons were identified based on well-established morphological criteria under light microscopy, including relatively large cell size, polygonal or multipolar shape, a prominent euchromatic nucleus with a visible nucleolus, and abundant eosinophilic cytoplasm (Rosado et al., 2017; Xiao et al., 2017). Histopathological analysis showed a significant decrease in neuron numbers in the ventral (Figure 5B) and dorsal (Figure 5C) horns of the spinal cord in the SCI group compared to the sham group (*p* < 0.001). SIL treatment, especially at 0.2 μmol, significantly increased neuron counts in both horns (*p* < 0.001).

### 3.4 Zymography results

**FIGURE 6:**
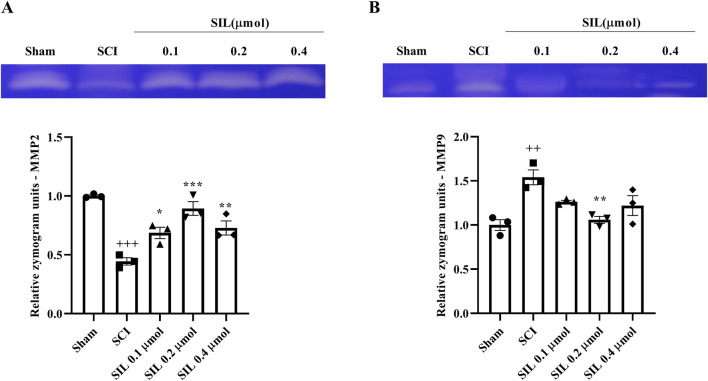
Effects of SIL on MMP2 (72 kDa) **(A)** and MMP9 (92 kDa) **(B)** levels following compression SCI. Data are expressed as mean ± SEM (*n* = 3). ^++^ *p* < 0.01, ^+++^ *p* < 0.001 vs. sham group and ^*^ *p* < 0.05, ^**^ *p* < 0.01, ^***^ *p* < 0.001 vs. SCI group. A one-way ANOVA was used. SCI: spinal cord injury; SIL: silymarin (0.1, 0.2, and 0.4 μmol, 10 μL, and 30 min post-SCI).

In addition, in this study, the levels of MMP2, as an anti-inflammatory enzyme, and MMP9, as an inflammatory enzyme, were evaluated. Following SCI, a significant decrease in MMP2 levels was observed (*p* < 0.001). However, treatment with SIL, specifically at a dose of 0.2 μmol, significantly increased MMP2 levels (*p* < 0.001) (Figure 6A) and suppressed MMP9 (*p* < 0.05) (Figure 6B).

### 3.5 Biochemical results: catalase, glutathione, and nitrite

#### 3.5.1 Catalase and glutathione levels

**FIGURE 7:**
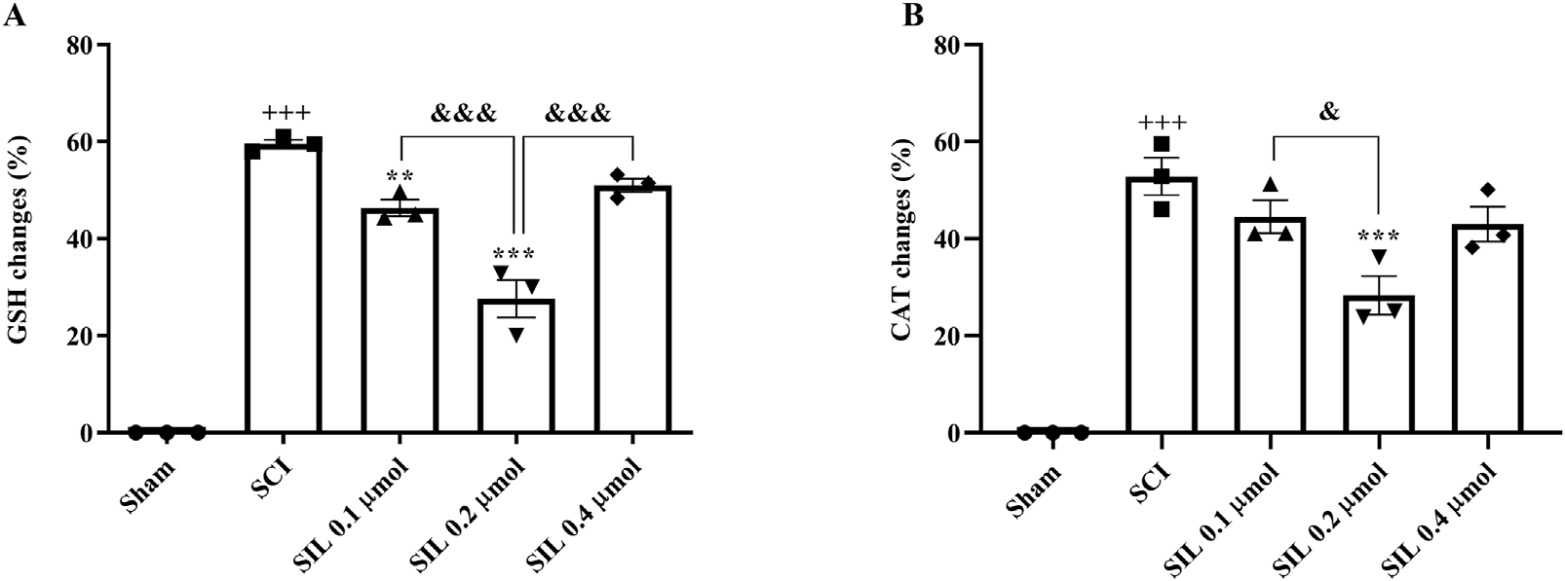
Effects of SIL on serum levels of GSH **(A)** and CAT **(B)** following compression SCI. Data are expressed as mean ± SEM (*n* = 3). ^+++^ *p* < 0.001 vs. sham group, ^***^ *p* < 0.001 vs. SCI group, and ^&^ *p* < 0.05, and ^&&&^ *p* < 0.001 vs. groups shown by lines. A one-way ANOVA was used. CAT, catalase; GSH, glutathione; SCI: spinal cord injury; SIL: silymarin (0.1, 0.2, and 0.4 μmol, 10 μL, and 30 min post-SCI).

The results showed that the changes in serum levels of glutathione (Figure 7A) and catalase (Figure 7B) in the SCI group were significant compared to the sham group (*p* < 0.001). Treatment with SIL significantly compensated for this decrease (*p* < 0.001).

#### 3.5.2 Nitrite level

**FIGURE 8:**
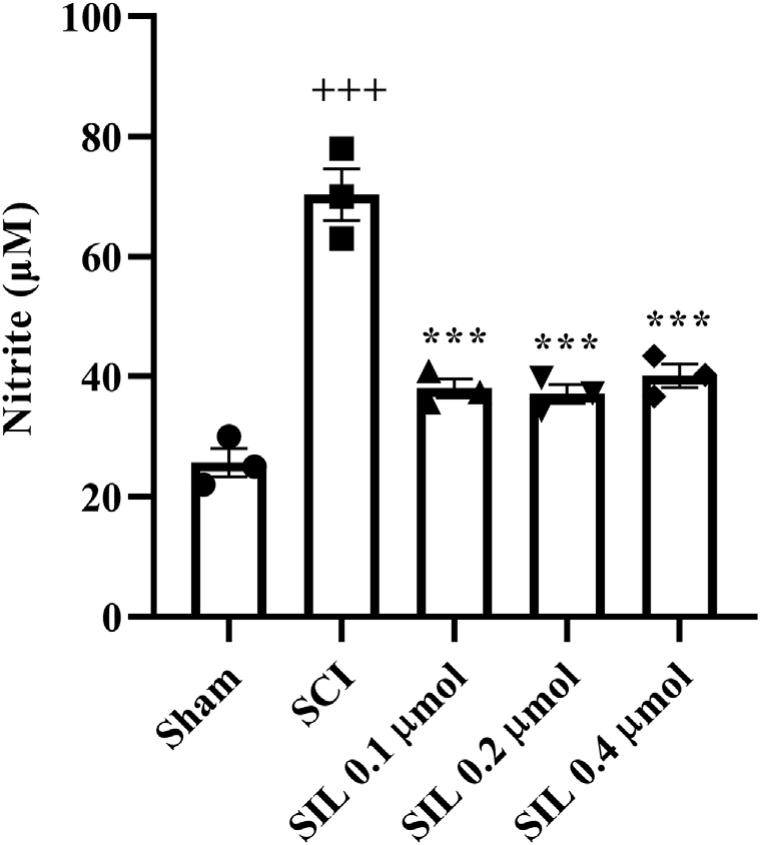
Effects of SIL on nitrite levels of serum following compression SCI. Data are expressed as mean ± SEM (*n* = 3). ^+++^ *p* < 0.001 vs. sham group and ^***^ *p* < 0.001 vs. SCI group. A one-way ANOVA was used. SCI: spinal cord injury; SIL: silymarin (0.1, 0.2, and 0.4 μmol, 10 μL, and 30 min post-SCI).

SCI increased the serum nitrite level in the SCI group compared to the sham group (*p* < 0.001). Treatment with SIL reduced these elevated levels (*p* < 0.001) (Figure 8).

## 4 Discussion

The data from this study indicate that i.t. administration of SIL is associated with dose-dependent improvements in functional outcomes following compression SCI. As shown in Figure 1, SIL treatment improved behavioral performance in assessments of motor function and pain sensitivity, reduced markers of oxidative stress, and modulated inflammatory mediators. Specifically, SIL attenuated cold allodynia, heat hyperalgesia, and mechanical allodynia, while improving motor coordination and mitigating SCI-induced weight loss. Biochemical analyses revealed enhanced activities of antioxidant enzymes such as catalase and glutathione, along with reduced serum nitrite levels. In addition, SIL treatment decreased levels of the pro-inflammatory enzyme MMP9 and increased levels of the anti-inflammatory MMP2. Histological findings further showed improved preservation of sensory and motor neurons in the dorsal and ventral horns of the spinal cord in SIL-treated animals.

The sensory and motor deficits following SCI depend largely on the extent and location of the injury, which can be classified according to the American Spinal Injury Association (ASIA) impairment scale into five categories: complete loss of motor and sensory function (A), sensory preserved without motor function (B), incomplete motor function (C and D), and normal function (E) (Holmes et al., 2024). Hence, in the current study, we used the compression SCI model to assess the impact of SIL translated to such an ASIA score of B (Fakhri et al., 2019; Fakhri et al., 2021; Fakhri et al., 2022b; Bagheri Bavandpouri et al., 2024). Research has identified various methods of inducing SCI models, with the compression SCI model being the most appropriate. This model applies pressure on both the dorsal and ventral surfaces of the spinal cord, allowing for an assessment of the sensory and motor pathway disruptions caused by SCI (Sharif-Alhoseini and Rahimi-Movaghar, 2014; Alizadeh et al., 2019).

The present study demonstrates that i.t. administration of SIL promotes functional recovery after compression SCI in a dose-dependent manner. Among the doses tested (0.1, 0.2, and 0.4 μmol), the 0.2 μmol dose consistently showed the most significant improvement across behavioral, biochemical, and histological assessments.

The effectiveness of SIL at a 0.2 μmol dose underscores the significance of identifying optimal dosing strategies. This finding suggests a dose-dependent relationship, indicating that higher doses do not necessarily yield enhanced effects but may lead to diminished efficacy or increased potential side effects (Peper, 2009). This type of dose-response efficacy was founded on a reverse U-shaped dose-response relationship and the principle of hormesis (Fakhri et al., 2020; Fakhri et al., 2022b). As a principle, the biphasic dose-response relationship, hormesis is a dose-response phenomenon well-known by a high-dose inhibition and low-dose stimulation of biological responses, represented by an inverted U-shaped dose-response or either a graphical J/U-shaped curve. Consequently, while high doses indicate possible cellular damage, decreased doses represent adaptive biological performance, including growth, cognition, longevity, and other biological activities (Fakhri et al., 2022b). It is also important to note that i.t. drug delivery allows for more immediate and potent effects compared to traditional oral routes. By bypassing the blood-brain barrier, medications can achieve higher concentrations directly at the target site in the central nervous system (CNS) without needing to navigate systemic circulation. This direct access results in improved therapeutic outcomes with potentially lower doses of medication (Bottros and Christo, 2014; De Andres et al., 2022). In addition to the importance of dose selection and drug delivery method, justification of drug solvent is also of great importance. Accordingly, we employed 70% ethanol to prepare the stock solution, as ethanol has a good ability to dissolve the contained flavonolignan compounds, and using this solvent ratio is appropriate to increase the solubility of the SIL (Tsai et al., 2010; Javed et al., 2011; Csupor et al., 2016). This dilution step (1/9: solution/distilled water: v/v) is very important because the final concentration of the solution must be safe and tolerable for i.t. injection and to prevent irritation or damage to the nervous. This volume is common for i.t. injections in animal models and allows suitable penetration of the compound into the central nervous system (Fakhri et al., 2022a; Rahman et al., 2023).

Behavioral evaluations revealed that SCI rats experienced significant motor deficits, as shown by lower BBB scores and impaired performance in the inclined plane test. SIL treatment significantly improved motor function from the early days post-injury, with the 0.2 μmol dose showing the most significant effect. Additionally, sensory abnormalities manifested as heat hyperalgesia, cold allodynia, and mechanical allodynia were notably attenuated by SIL, further confirming its analgesic properties. These findings align well with previous reports demonstrating the neuroprotective and anti-neuropathic effects of SIL in various neurodegenerative and neuropathic pain models (Haddadi et al., 2014; Hassani et al., 2015; de Freitas et al., 2018; Mohajjel Nayebi et al., 2021).

Oxidative stress is a key contributor to neuropathic pain and motor dysfunction after SCI through mechanisms involving inflammation, nerve damage, and sensitization of pain pathways (Singh et al., 2019; Hendrix et al., 2020; Yu et al., 2023). Accordingly, the attenuation of oxidative damage by SIL provided a plausible mechanism underlying the functional recovery seen in our study. When the body’s capacity to neutralize or detoxify ROS and reactive nitrogen species (RNS) is imbalanced, oxidative stress results. Highly reactive molecules called ROS have the potential to harm DNA, lipids, and proteins as well as other cellular components. This damage can lead to inflammation and dysfunction of cells and tissues (Singh et al., 2019), such as the process that happens after SCI (Yu et al., 2023). SIL is a flavonoid complex derived from the milk thistle plant. SIL has been found to possess antioxidant, anti-inflammatory, and neuroprotective properties (Surai, 2015). Also, Asghar et al. demonstrated that SIL possesses potent antioxidant properties by directly scavenging free radicals, inhibiting ROS-generating enzymes, enhancing cellular antioxidant defenses, and suppressing peroxyl radicals (Asghar and Masood, 2008). Furthermore, our biochemical data demonstrating increased catalase and glutathione peroxidase activity, along with decreased serum nitrite levels, reinforce the role of oxidative stress modulation in the sensory and motor improvements induced by SIL.

Nitric oxide and oxidative stress can interact with each other and affect various pathways, such as inflammation, apoptosis, and autophagy (Kumar and Chanana, 2017). Nitric oxide can affect antioxidant enzyme activity (SOD and CAT) and regulate the expression of genes related to the oxidative stress response (Kumar and Chanana, 2017). Oral administration of SIL in the Alzheimer’s model was associated with a decrease in NO and malondialdehyde (MDA) levels and an increase in the activity of SOD and CAT, as well as a decrease in the inflammatory factors TNF-α and IL-1β, improvement in histological changes, and preservation of neurons (Begara-Morales et al., 2016). Silibinin, another compound of milk thistle, has been studied for its potential to alleviate ferroptosis, a type of non-apoptotic cell death associated with SCI. Research indicates that it is effective in decreasing iron accumulation and lipid peroxidation, as evidenced by reduced levels of MDA, while simultaneously promoting increases in glutathione, glutathione peroxidase-4, and ferroportin levels (Vahabi et al., 2024). Tsai et al. highlighted the beneficial effects of SIL and silybin on cultured cells and in cases of acute SCI. Both compounds exhibit antioxidative, anti-inflammatory, and free radical scavenging properties, which may contribute to the prevention or mitigation of neurodegenerative diseases. They reported that SIL seems to be more effective than silybin in reducing free radical levels (Tsai et al., 2010).

ROS activate inflammatory signaling pathways (e.g., NF-κB), increasing the production of pro-inflammatory mediators. Inflammation generates more ROS through immune cell activation, perpetuating oxidative damage. This vicious cycle amplifies secondary injury, neuronal loss, and functional deficits (Yin et al., 2024). Following inflammation, pro-inflammatory cytokines (e.g., TNF-α, IL-1β) and enzymes such as MMPs are released, which further aggravate tissue injury by promoting edema, blood-spinal cord barrier disruption, and neuronal apoptosis (Freyermuth-Trujillo et al., 2022). In our study, SCI resulted in decreased levels of anti-inflammatory MMP2 and increased pro-inflammatory MMP9. SIL treatment reversed these changes by increasing MMP2 and suppressing MMP9 activities. A study reported that SIL reduces cyclooxygenase-2 (COX-2) expression and modulates the levels of NF-κB and MMPs (Ullah and Khan, 2018). Also, another study mentioned that SIL modulates the expression and activity of MMPs (Wadhwa et al., 2022). In the context of SCI, we previously showed that MMP9 plays an inflammatory role, while MMP2 possesses anti-inflammatory potentials (Bagheri Bavandpouri et al., 2024).

After the administration of SIL following SCI, the histology results and the increase in the number of healthy neurons align with the improvement observed in sensory and motor function. Aboelwafa et al. demonstrated that SIL significantly enhances neuronal integrity in animal models exposed to neurotoxic agents, such as aluminum chloride (Aboelwafa et al., 2020). Our findings suggested that SIL can potentially facilitate the repair of sensory and motor neurons in the dorsal and ventral horns of the spinal cord after SCI.

During the initial stage of SCI, individuals often experience substantial weight loss because of metabolic alterations and muscle degradation (Giangregorio and McCartney, 2006). Our study showed that SIL enhanced the motor activity of SCI rats by effectively alleviating pain, subsequently increasing their appetite and restoring their regular food consumption. By supporting liver function, SIL may help enhance overall metabolism and improve weight management (Vahabzadeh et al., 2018; Gillessen and Schmidt, 2020).

Despite using the most clinically relevant models of SCI in the current study (e.g., compression), there are minor limitations in related animal models. In pre-clinical models of SCI, young adult animals are commonly employed, while a wide age range of human individuals is involved in clinical models. Larger gray matter volumes in humans cause some neural tracts to be affected in human models and slow recovery in humans after SCI (Bagheri Bavandpouri et al., 2024).

## 5 Conclusion

The findings from this study highlight the significant therapeutic potential of i.t. administration of SIL in a dose-dependent manner in facilitating sensory-functional recovery after compression SCI. SIL exerts its beneficial effects through a variety of effects, including its antioxidant, anti-inflammatory, and neuroprotective properties, which collectively help to counteract the harmful consequences of oxidative stress and inflammation commonly associated with SCI. Nevertheless, further investigation is warranted to fully elucidate the specific molecular mechanistic pathways through which SIL exerts its effects, followed by well-controlled clinical trials.

## Data Availability

The raw data supporting the conclusions of this article will be made available by the authors, without undue reservation.

## Abbreviations

Akt, Protein kinase B; BBB, Basso, Beattie, and Bresnahan; COX-2, Cyclooxygenase-2; DTNB, dithiobisnitrobenzoic acid; H&E, Hematoxylin and eosin; IL-1β, Interleukin-1beta; i.t., Intrathecal; JNK, C-Jun N-terminal kinase; MAPK, Mitogen-activated protein kinase; MMP2, Matrix metalloproteinase 2; mTOR, Mammalian target of rapamycin; MDA, Malondialdehyde; NF-κB, Nuclear factor-kappa B; NO, Nitric oxide; ROS, Reactive oxygen species; RNS, Reactive nitrogen species; SIL, Silymarin; SCI, Spinal cord injury.

## References

[B1] Aboelwafa H. R. El-Kott A. F. Abd-Ella E. M. Yousef H. N. (2020). The possible neuroprotective effect of silymarin against aluminum chloride-prompted alzheimer’s-like disease in rats. Brain Sci. 10, 628. 10.3390/brainsci10090628 32932753 PMC7564174

[B2] AbouZid S. F. Chen S.-N. Pauli G. F. (2016). Silymarin content in Silybum marianum populations growing in Egypt. Ind. Crops Prod. 83, 729–737. 10.1016/j.indcrop.2015.12.012 27182123 PMC4863705

[B3] Aebi H. (1984). Catalase *in vitro* . Methods Enzym. 105, 121–126. 10.1016/S0076-6879(84)05016-3 6727660

[B4] Alizadeh A. Dyck S. M. Karimi-Abdolrezaee S. (2019). Traumatic spinal cord injury: an overview of pathophysiology, models and acute injury mechanisms. Front. Neurol. 10, 282. 10.3389/fneur.2019.00282 30967837 PMC6439316

[B5] Amirian R. Mohammadi Pour P. Maleki H. Fakhri S. Asgary S. Farzaei M. H. (2025). Evaluating the anti-neuropathic effects of the thymol-loaded nanofibrous scaffold in a rat model of spinal cord injury. Front. Pharmacol. 16, 1507397. 10.3389/fphar.2025.1507397 40255564 PMC12006068

[B6] Asghar Z. Masood Z. (2008). Evaluation of antioxidant properties of silymarin and its potential to inhibit peroxyl radicals *in vitro* . Pak. J. Pharm. Sci. 21, 249–254. Available online at: http://www.ncbi.nlm.nih.gov/pubmed/18614420. 18614420

[B7] Bagheri Bavandpouri F. S. Azizi A. Abbaszadeh F. Kiani A. Farzaei M. H. Mohammadi-Noori E. (2024). Polydatin attenuated neuropathic pain and motor dysfunction following spinal cord injury in rats by employing its anti-inflammatory and antioxidant effects. Front. Pharmacol. 15, 1452989. 10.3389/fphar.2024.1452989 39193334 PMC11347411

[B8] Begara-Morales J. C. Sánchez-Calvo B. Chaki M. Valderrama R. Mata-Pérez C. Padilla M. N. (2016). Antioxidant systems are regulated by nitric oxide-mediated post-translational modifications (NO-PTMs). Front. Plant Sci. 7, 152. 10.3389/fpls.2016.00152 26909095 PMC4754464

[B9] Bottros M. M. Christo P. J. (2014). Current perspectives on intrathecal drug delivery. J. Pain Res. 7, 615–626. 10.2147/JPR.S37591 25395870 PMC4227625

[B10] Csupor D. Csorba A. Hohmann J. (2016). Recent advances in the analysis of flavonolignans of Silybum marianum. J. Pharm. Biomed. Anal. 130, 301–317. 10.1016/j.jpba.2016.05.034 27321822

[B11] De Andres J. Hayek S. Perruchoud C. Lawrence M. M. Reina M. A. De Andres-Serrano C. (2022). Intrathecal drug delivery: advances and applications in the management of chronic pain patient. Front. pain Res. Lausanne, Switz. 3, 900566. 10.3389/fpain.2022.900566 35782225 PMC9246706

[B12] de Freitas C. M. Krum B. N. Chiapinotto Ceretta A. P. Schaffer L. F. de Moraes Reis E. Schwerz J. P. (2018). Silymarin recovers 6-hydroxydopamine-induced motor deficits in mice. Food Chem. Toxicol. 118, 549–556. 10.1016/j.fct.2018.05.062 29852213

[B13] Eyer P. Podhradský D. (1986). Evaluation of the micromethod for determination of glutathione using enzymatic cycling and Ellman’s reagent. Anal. Biochem. 153, 57–66. 10.1016/0003-2697(86)90061-8 3963383

[B14] Fakhri S. Dargahi L. Abbaszadeh F. Jorjani M. (2018). Astaxanthin attenuates neuroinflammation contributed to the neuropathic pain and motor dysfunction following compression spinal cord injury. Brain Res. Bull. 143, 217–224. 10.1016/j.brainresbull.2018.09.011 30243665

[B15] Fakhri S. Dargahi L. Abbaszadeh F. Jorjani M. (2019). Effects of astaxanthin on sensory‐motor function in a compression model of spinal cord injury: involvement of ERK and AKT signalling pathway. Eur. J. Pain 23, 750–764. 10.1002/ejp.1342 30427581

[B16] Fakhri S. Ahmadpour Y. Rezaei H. Kooshki L. Moradi S. Z. Iranpanah A. (2020). The antinociceptive mechanisms of melatonin: role of L-arginine/nitric oxide/cyclic GMP/KATP channel signaling pathway. Behav. Pharmacol. 31, 728–737. 10.1097/FBP.0000000000000579 32925224

[B17] Fakhri S. Kiani A. Jalili C. Abbaszadeh F. Piri S. Farzaei M. H. (2021). Intrathecal administration of melatonin ameliorates the neuroinflammation- mediated sensory and motor dysfunction in A rat model of compression spinal cord injury. Curr. Mol. Pharmacol. 14, 646–657. 10.2174/1874467213666201230101811 33380311

[B18] Fakhri S. Abbaszadeh F. Dargahi L. Pouriran R. Jorjani M. (2022a). Astaxanthin ameliorates serum level and spinal expression of macrophage migration inhibitory factor following spinal cord injury. Behav. Pharmacol. 33, 505–512. 10.1097/FBP.0000000000000698 36148838

[B19] Fakhri S. Piri S. Moradi S. Z. Khan H. (2022b). Phytochemicals targeting oxidative stress, interconnected neuroinflammatory, and neuroapoptotic pathways following radiation. Curr. Neuropharmacol. 20, 836–856. 10.2174/1570159X19666210809103346 34370636 PMC9881105

[B20] Fakhri S. Sabouri S. Kiani A. Farzaei M. H. Rashidi K. Mohammadi-Farani A. (2022c). Intrathecal administration of naringenin improves motor dysfunction and neuropathic pain following compression spinal cord injury in rats: relevance to its antioxidant and anti-inflammatory activities. Korean J. Pain 35, 291–302. 10.3344/kjp.2022.35.3.291 35768984 PMC9251389

[B21] Freire M. A. M. Rocha G. S. Bittencourt L. O. Falcao D. Lima R. R. Cavalcanti J. R. L. P. (2023). Cellular and molecular pathophysiology of traumatic brain injury: what have we Learned so far? Biol. (Basel) 12, 1139. 10.3390/biology12081139 37627023 PMC10452099

[B22] Freyermuth-Trujillo X. Segura-Uribe J. J. Salgado-Ceballos H. Orozco-Barrios C. E. Coyoy-Salgado A. (2022). Inflammation: a target for treatment in spinal cord injury. Cells 11, 2692. 10.3390/cells11172692 36078099 PMC9454769

[B23] Gensel J. C. Zhang B. (2015). Macrophage activation and its role in repair and pathology after spinal cord injury. Brain Res. 1619, 1–11. 10.1016/j.brainres.2014.12.045 25578260

[B24] Giangregorio L. McCartney N. (2006). Bone loss and muscle atrophy in spinal cord injury: epidemiology, fracture prediction, and rehabilitation strategies. J. Spinal Cord. Med. 29, 489–500. 10.1080/10790268.2006.11753898 17274487 PMC1949032

[B25] Gillessen A. Schmidt H. H.-J. (2020). Silymarin as supportive treatment in liver diseases: a narrative review. Adv. Ther. 37, 1279–1301. 10.1007/s12325-020-01251-y 32065376 PMC7140758

[B26] Guo H. Cao H. Cui X. Zheng W. Wang S. Yu J. (2019). Silymarin’s inhibition and treatment effects for alzheimer’s disease. Molecules 24, 1748. 10.3390/molecules24091748 31064071 PMC6539875

[B27] Haddadi R. Nayebi A. M. Farajniya S. Brooshghalan S. E. Sharifi H. (2014). Silymarin improved 6-OHDA-induced motor impairment in hemi-parkisonian rats: behavioral and molecular study. Daru 22, 38. 10.1186/2008-2231-22-38 24726284 PMC4001109

[B28] Hadi A. Pourmasoumi M. Mohammadi H. Symonds M. Miraghajani M. (2018). The effects of silymarin supplementation on metabolic status and oxidative stress in patients with type 2 diabetes mellitus: a systematic review and meta-analysis of clinical trials. Complement. Ther. Med. 41, 311–319. 10.1016/j.ctim.2018.08.010 30477860

[B29] Hassani F. V. Rezaee R. Sazegara H. Hashemzaei M. Shirani K. Karimi G. (2015). Effects of silymarin on neuropathic pain and formalin-induced nociception in mice. Iran. J. Basic Med. Sci. 18, 715–720. Available online at: http://www.ncbi.nlm.nih.gov/pubmed/26351564. 26351564 PMC4556767

[B30] Hendrix J. Nijs J. Ickmans K. Godderis L. Ghosh M. Polli A. (2020). The interplay between oxidative stress, exercise, and pain in health and disease: potential role of autonomic regulation and epigenetic mechanisms. Antioxidants Basel, Switz. 9, 1166. 10.3390/antiox9111166 33238564 PMC7700330

[B31] Holmes B. D. Brazauskas R. Chhabra H. S. (2024). Spinal cord injury etiology, severity, and care in east asia: a cross-sectional analysis of the international spinal cord society database Project. Spinal Cord. 62, 421–427. 10.1038/s41393-024-01003-7 38914754

[B32] Huang W. L. King V. R. Curran O. E. Dyall S. C. Ward R. E. Lal N. (2007). A combination of intravenous and dietary docosahexaenoic acid significantly improves outcome after spinal cord injury. Brain 130, 3004–3019. 10.1093/brain/awm223 17901087

[B33] Javed S. Kohli K. Ali M. (2011). Reassessing bioavailability of silymarin. Altern. Med. Rev. 16, 239–249. Available online at: http://www.ncbi.nlm.nih.gov/pubmed/21951025. 21951025

[B34] Jiang C. Chen Z. Wang X. Zhang Y. Guo X. Fan H. (2023). Curcumin-activated olfactory ensheathing cells improve functional recovery after spinal cord injury by modulating microglia polarization through APOE/TREM2/NF-κB signaling pathway. J. Neuroimmune Pharmacol. 18, 476–494. 10.1007/s11481-023-10081-y 37658943 PMC10577109

[B35] Kauppila T. (2000). Cold exposure enhances tactile allodynia transiently in mononeuropathic rats. Exp. Neurol. 161, 740–744. 10.1006/exnr.1999.7287 10686093

[B36] Koltai T. Fliegel L. (2022). Role of silymarin in cancer treatment: facts, hypotheses, and questions. J. evidence-based Integr. Med. 27, 2515690X211068826. 10.1177/2515690X211068826 35018864 PMC8814827

[B37] Kumar A. Chanana P. (2017). Role of nitric oxide in stress-induced anxiety: from pathophysiology to therapeutic target. Vitam. Horm. 103, 147–167. 10.1016/bs.vh.2016.09.004 28061969

[B38] Kwak E. K. Kim J. W. Kang K. S. Lee Y. H. Hua Q. H. Park T. I. (2005). The role of inducible nitric oxide synthase following spinal cord injury in rat. J. Korean Med. Sci. 20, 663–669. 10.3346/jkms.2005.20.4.663 16100462 PMC2782166

[B39] Liu X. Zhang Y. Wang Y. Qian T. (2021). Inflammatory response to spinal cord injury and its treatment. World Neurosurg. 155, 19–31. 10.1016/j.wneu.2021.07.148 34375779

[B40] Mestre C. Pélissier T. Fialip J. Wilcox G. Eschalier A. (1994). A method to perform direct transcutaneous intrathecal injection in rats. J. Pharmacol. Toxicol. Methods 32, 197–200. 10.1016/1056-8719(94)90087-6 7881133

[B41] Mohajjel Nayebi A. Hashemian A. Rezazadeh H. Charkhpour M. Fekri K. Haddadi R. (2021). Silymarin reduced cisplatin-induced hyperalgesia bysuppressing oxidative stress in male rats. Physiol. Pharmacol. 25, 146–153. 10.32598/ppj.25.2.60

[B42] Peper A. (2009). Aspects of the relationship between drug dose and drug effect. Dose. Response 7, 172–192. 10.2203/dose-response.08-019.Peper 19543483 PMC2695574

[B43] Rahman M. M. Lee J. Y. Kim Y. H. Park C.-K. (2023). Epidural and intrathecal drug delivery in rats and mice for experimental research: fundamental Concepts, Techniques, precaution, and application. Biomedicines 11, 1413. 10.3390/biomedicines11051413 37239084 PMC10216595

[B44] Rosado I. R. Carvalho P. H. Alves E. G. L. Tagushi T. M. Carvalho J. L. Silva J. F. (2017). Immunomodulatory and neuroprotective effect of cryopreserved allogeneic mesenchymal stem cells on spinal cord injury in rats. Genet. Mol. Res. 16. 10.4238/gmr16019555 28340277

[B45] Samini F. Samarghandian S. Borji A. Mohammadi G. Bakaian M. (2013). Curcumin pretreatment attenuates brain lesion size and improves neurological function following traumatic brain injury in the rat. Pharmacol. Biochem. Behav. 110, 238–244. 10.1016/j.pbb.2013.07.019 23932920

[B46] Sharif-Alhoseini M. Rahimi-Movaghar V. (2014). “Animal models in traumatic spinal cord injury,” in Topics in paraplegia (Zagreb: InTech). 10.5772/57189

[B47] Singh A. Kukreti R. Saso L. Kukreti S. (2019). Oxidative stress: a key modulator in neurodegenerative diseases. Molecules 24, 1583. 10.3390/molecules24081583 31013638 PMC6514564

[B48] Singla R. K. Singh D. Verma R. Kaushik D. Echeverría J. Garg V. (2024). Fermented formulation of Silybum marianum seeds: Optimization, heavy metal analysis, and hepatoprotective assessment. Phytomedicine 124, 155286. 10.1016/j.phymed.2023.155286 38241906

[B49] Sun J. Zhang X. Broderick M. Fein H. (2003). Measurement of nitric oxide production in biological systems by using Griess reaction assay. Sensors 3, 276–284. 10.3390/s30800276

[B50] Surai P. F. (2015). Silymarin as a natural antioxidant: an overview of the current evidence and perspectives. Antioxidants Basel, Switz. 4, 204–247. 10.3390/antiox4010204 26785346 PMC4665566

[B51] Tsai M.-J. Liao J.-F. Lin D.-Y. Huang M.-C. Liou D.-Y. Yang H.-C. (2010). Silymarin protects spinal cord and cortical cells against oxidative stress and lipopolysaccharide stimulation. Neurochem. Int. 57, 867–875. 10.1016/j.neuint.2010.09.005 20868716

[B52] Ullah H. Khan H. (2018). Anti-Parkinson potential of silymarin: mechanistic Insight and therapeutic standing. Front. Pharmacol. 9, 422. 10.3389/fphar.2018.00422 29755356 PMC5934474

[B53] Vahabi A. Öztürk A. M. Kılıçlı B. Birim D. Kaftan Öcal G. Dağcı T. (2024). Silibinin promotes healing in spinal cord injury through anti-ferroptotic mechanisms. JOR spine 7, e1344. 10.1002/jsp2.1344 38957164 PMC11217020

[B54] Vahabzadeh M. Amiri N. Karimi G. (2018). Effects of silymarin on metabolic syndrome: a review. J. Sci. Food Agric. 98, 4816–4823. 10.1002/jsfa.9115 29736939

[B55] Wadhwa K. Pahwa R. Kumar M. Kumar S. Sharma P. C. Singh G. (2022). Mechanistic Insights into the Pharmacological significance of silymarin. Molecules 27, 5327. 10.3390/molecules27165327 36014565 PMC9414257

[B56] Wang C. Wang Z. Zhang X. Zhang X. Dong L. Xing Y. (2012). Protection by silibinin against experimental ischemic stroke: up-regulated pAkt, pmTOR, HIF-1α and Bcl-2, down-regulated Bax, NF-κB expression. Neurosci. Lett. 529, 45–50. 10.1016/j.neulet.2012.08.078 22999929

[B57] Xiao W. Wen J. Huang Y.-C. Yu B.-S. (2017). Development of a modified model of spinal cord ischemia injury by selective ligation of lumbar arteries in rabbits. Spinal Cord. 55, 1028–1032. 10.1038/sc.2017.66 28607524

[B58] Yin Z. Wan B. Gong G. Yin J. (2024). ROS: Executioner of regulating cell death in spinal cord injury. Front. Immunol. 15, 1330678. 10.3389/fimmu.2024.1330678 38322262 PMC10844444

[B59] Yu M. Wang Z. Wang D. Aierxi M. Ma Z. Wang Y. (2023). Oxidative stress following spinal cord injury: from molecular mechanisms to therapeutic targets. J. Neurosci. Res. 101, 1538–1554. 10.1002/jnr.25221 37272728

[B60] Zhang H. Chang M. Hansen C. N. Basso D. M. Noble-Haeusslein L. J. (2011). Role of matrix metalloproteinases and therapeutic Benefits of their inhibition in spinal cord injury. Neurotherapeutics 8, 206–220. 10.1007/s13311-011-0038-0 21455784 PMC3077748

[B61] Zhang Y. Liu Z. Zhang W. Wu Q. Zhang Y. Liu Y. (2019). Melatonin improves functional recovery in female rats after acute spinal cord injury by modulating polarization of spinal microglial/macrophages. J. Neurosci. Res. 97, 733–743. 10.1002/jnr.24409 31006904

